# Lessons from a community vaccination programme to control a meningococcal disease serogroup W outbreak in remote South Australia, 2017

**DOI:** 10.5365/wpsar.2019.10.2.002

**Published:** 2021-03-08

**Authors:** Louise Flood, Matthew McConnell, Luda Molchanoff, Zell Dodd, Jana Sisnowski, Melissa Fidock, Tina Miller, Karli Borresen, Hannah Vogt, Andrew Lane

**Affiliations:** a Communicable Disease Control Branch, South Australia Department for Health and Wellbeing, Adelaide, South Australia, Australia.; b Country Health SA Local Health Network, South Australia Department for Health and Wellbeing, Adelaide, South Australia, Australia.; c Ceduna Koonibba Aboriginal Health Service, Ceduna, South Australia, Australia.; d Media and Communications Branch, South Australia Department for Health and Wellbeing, Adelaide, South Australia, Australia.

## Abstract

**Problem:**

From December 2016 to February 2017, two cases of invasive meningococcal disease and one case of meningococcal conjunctivitis, all serogroup W, occurred in Aboriginal children in the Ceduna region of South Australia. The clustering of cases in time and place met the threshold for a community outbreak.

**Context:**

The Ceduna region is a remote part of South Australia, with more than 25% of the population identifying as Aboriginal or Torres Strait Islander.

**Action:**

As part of the outbreak response, a community-wide meningococcal vaccination programme against serogroups A, C, W and Y was implemented in a collaboration among different agencies of the South Australia Department for Health and Well-being, Aboriginal health and community services providers, and other local service providers and government agencies. The programme comprised an outbreak vaccination schedule, targeting all people aged ^3^ 2 months residing in the cases’ places of residence or in towns with close links.

**Outcome:**

Between March and June 2017, 3383 persons were vaccinated, achieving an estimated coverage of 71–85% of the target population, with 31% (*n* = 1034) of those vaccinated identifying as Aboriginal or Torres Strait Islander. No local cases of serogroup W occurred during the vaccination programme, but two further cases were notified by the end of 2018.

**Discussion:**

The participation of a large number of local and non-health-sector stakeholders in programme planning and implementation, a clear response management structure and high community acceptability were identified as key factors that contributed to the programme achieving high vaccination coverage. The need to develop standard operating procedures for community-based outbreak response interventions to ease logistical challenges was considered an important lesson learnt.

*Neisseria meningitidis* is a Gram-negative diplococcus and the causative agent of invasive meningococcal disease (IMD). IMD commonly presents with meningitis and septicaemia. (*1*, *2*) Long-term sequelae may include limb amputation, hearing loss and neurological impairment. (*2*) Six serogroups account for nearly all human cases globally; (*1*) in some reports, serogroup W is associated with higher case fatality rates and more frequent atypical presentations. (*3*, *4*) Worldwide, an estimated 10–20% of people asymptomatically carry *N. meningitidis* in their upper respiratory tract, (*1*) with the highest carriage rates found in adolescents and young adults. (*5*)

IMD is a notifiable disease in all Australian jurisdictions. Meningococcal conjunctivitis may precede IMD in cases or contacts and is usually notified. (*6*) Nationally, the epidemiology of IMD has changed markedly in the past several years, with serogroup W replacing serogroup B as the most common serogroup since 2016. (*7*) By contrast, in South Australia (SA), serogroup B was responsible for 81% (22/27) of notifications in 2016 and serogroup W for the remainder. Compared with non-Indigenous Australians, Aboriginal and Torres Strait Islander people have higher rates of IMD, particularly serogroup W. (*8*)

Several meningococcal vaccines against serogroups A, C, W and Y are available for private purchase in Australia and have been funded under the National Immunization Program from July 2018 for infants and April 2019 for adolescents.

## Problem

From December 2016 to February 2017, the Communicable Disease Control Branch at the SA Department for Health and Well-being (SA Health) was notified of two cases of IMD serogroup W and one case of meningococcal conjunctivitis serogroup W in the Ceduna Local Government Area. Serogroup W had not been notified in this region since records started in 1990. All three cases occurred in Aboriginal children aged 2 to 12 years, with no additional epidemiological links between the cases. Fine typing was available for two of the three cases: both were P1.5,2:F1–1. As part of routine public health follow-up of sporadic cases, the Communicable Disease Control Branch directed that close contacts should receive clearance antibiotics, and approximately 300 contacts, including close contacts, were vaccinated in January and February 2017.

The Ceduna Local Government Area is a remote part of Australia, with an estimated resident population of 3716 persons as of 30 June 2016. Approximately 25% of residents identify as Aboriginal or Torres Strait Islander. The estimated attack rate of 81 cases (or 54 invasive cases) per 100 000 population during the three months from December 2016 through February 2017 exceeded not only the threshold for defining a community outbreak of 10 cases per 100 000 population as defined by the National Guidelines of the Communicable Diseases Network Australia, but also the lower thresholds for implementing population-wide disease control measures in remote Aboriginal or Torres Strait Islander communities. (*6*) An outbreak response was commenced, and a community-wide vaccination programme was implemented to prevent the occurrence of further cases of IMD serogroup W in the Ceduna region.

## Action

### Programme design and setting

At the time of programme inception and implementation, publicly funded health services in regional and remote SA were provided by the Country Health SA Local Health Network (CHSALHN), which was part of SA Health. In addition, Aboriginal Community Controlled Health Services operate across SA. Multiple national, state and local organizations were involved in planning and implementing the vaccination programme (**Box 1**).

Box 1 **Organizations involved in planning and implementing the Ceduna community vaccination programme, South Australia, 2017**

**Commonwealth (national), state and local government entities**

- Commonwealth Department of the Prime Minister and Cabinet, Ceduna Office
- SA Health, including:
- Country Health SA Local Health Network, Eyre and Far North Region and Corporate Office
- Communicable Disease Control Branch
- Media and Communications Branch

- SA Ambulance Service

- SA Department for Child Protection,  Ceduna Office

- SA Department for Communities and  Social Inclusion, Housing SA and Ceduna Street Beat
- District Council of Ceduna

Aboriginal health and community services

- Ceduna Koonibba Aboriginal Health Service
- Tullawon Health Services, Yalata
- Oak Valley Health Services, Maralinga Tjarutja lands
- Aboriginal Health Council of South Australia
- Pangula Mannamurna Aboriginal Corporation
- Nunkuwarrin Yunti of South Australia Inc.

Other community services

- Centacare Catholic Family Services, Ceduna Office

A steering committee was convened to coordinate the outbreak response and was composed of representatives from the Communicable Disease Control Branch, CHSALHN and the Media and Communications Branch of SA Health; the Aboriginal Health Council of South Australia; and Ceduna Koonibba Aboriginal Health Service.

### Target population

**Figure 1:**
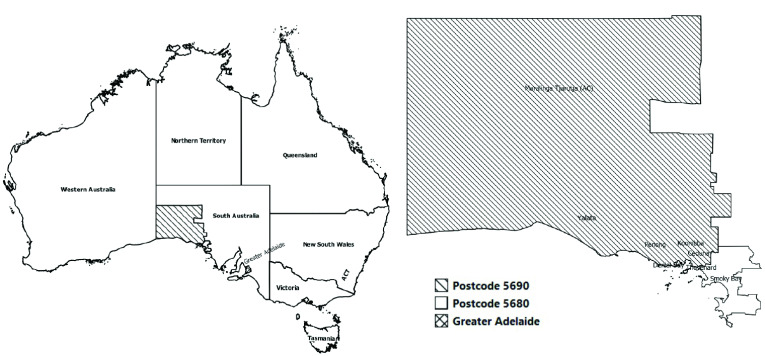
Map of the programme target area for vaccination with meningococcal ACWY vaccine including Australian Bureau of Statistics postal areas, state suburbs and the Maralinga Tjarutja Aboriginal Council (AC) Local Government Area, South Australia, 2017

Based on cases’ residence and known links between towns, the programme area (**Fig. 1**) encompassed Ceduna, Thevenard, Denial Bay, Koonibba, Yalata, Penong, Oak Valley in the Maralinga Tjarutja lands (lands owned by the Aboriginal traditional owners and administered as an Aboriginal Council, or AC), the homeland property Scotdesco (all of these are in postcode area 5690) and Smoky Bay (part of postcode area 5680). Given a lack of knowledge of meningococcal W carriage rates and the likely extent of population mixing, all Aboriginal and non-Aboriginal persons aged (*3*) 2 months were targeted for vaccination (meningococcal ACWY vaccines are not licensed for individuals aged < 2 months). Based on numbers from the Australian Bureau of Statistics and local records, eligibility for vaccination was estimated at 4000–4500 individuals.

[**insert** Figure 1]

### Vaccination schedule

The dosing schedule recommended in the *Australian Immunization Handbook* for persons travelling to epidemic-prone areas or mass gatherings (*9*) was used, that is, a primary vaccination course consisting of one to three doses, depending on the vaccine, age of the individual and their medical risk factors. At the time, Menveo^®^ (GlaxoSmithKline) was the only vaccine registered for use in infants younger than 12 months and was used to vaccinate children aged 2 months to < 12 months. Nimenrix^®^ (Pfizer) was originally intended to be used in all persons aged (*3*) 12 months because only one dose is required for all age groups in the absence of medical risk factors. However, due to limited vaccine supply following the concomitant introduction of adolescent meningococcal ACWY vaccination programmes in other Australian states, the vaccination schedule was altered to allow either Nimenrix or Menveo to be used in persons aged (*3*) 2 years. Because two doses of Menveo are required in children aged 12–23 months, Nimenrix was used exclusively in this age group.

### Resources

To staff vaccination clinics, additional clinical staff were made available from Aboriginal Health Services, other CHSALHN sites and regions, and from metropolitan areas. Other government and nongovernmental organizations contributed non-clinical staff. Standing medication orders for administering Menveo and Nimenrix had to be signed by each participating service.

A communication campaign was developed and implemented within two weeks and delivered for less than 2000 Australian dollars. Paid communications included a Facebook post, a local newspaper advertisement and a radio advertisement in English and Pitjantjatjara (the local Aboriginal language). Posters and fact sheets were created for both the public and health-care workers, and three press releases featuring local spokespersons targeted local and state newspapers. All information was made available centrally on the SA Health web site.

In addition to developing the schedule and standing medication orders for both vaccines, an immunization screening and consent form and a separate consent resource were developed for use on immunization day. Programme data were entered into a database, and the vaccines administered were retrospectively entered onto the Australian Immunization Register for patients whose Medicare numbers had been collected.

### Ethics statement

This article describes public health actions undertaken as part of an outbreak response under the South Australian Public Health Act 2011 that did not require ethics approval.

## Outcomes

The community vaccination programme commenced on 6 March 2017 and ran for two weeks at the Ceduna Town Hall. It continued until 30 June 2017 at Penong Town Hall (and included residents of Scotdesco), the Koonibba clinic, the Smoky Bay and Districts Community Club, the Tullawon Health Services Clinic at Yalata, the Oak Valley Health Clinic, the Ceduna Koonibba Aboriginal Health Service and the Ceduna Family Medical Practice. A total of 3383 individuals received a meningococcal ACWY vaccination, with 87 individuals recorded as requiring follow-up vaccination due to their age or medical risk status. No serious side-effects were reported. Data completeness exceeded 98% for the categories of Indigenous status, gender and age. Of those vaccinated, 52% (*n* = 1757) were female; 31% (*n* = 1034) identified as Aboriginal or Torres Strait Islander; and 91% (*n* = 3082) lived in a target suburb or one of the two postcodes containing those suburbs. The median age was 37 years (interquartile range: 17–55 years). Inclusive of the contacts of the first two cases, the programme reached almost 3700 people, estimated to represent 71–85% of the target population (**Table 1**).

**Table 1:** Number and overall coverage estimates of meningococcal ACWY vaccination by suburb and postcode, South Australia, 2017

Location	Vaccination events (*n*)	Population denominator^a^	Estimated coverage
**Total No. in target suburbs and case contacts^b^**	**3180**	**4000–4500**	**71–80%**
Ceduna	1584		
Thevenard	352		
Denial Bay	89		
Koonibba	129		
Smoky Bay	182		
Yalata	315		
Oak Valley	69		
Scotdesco	24		
Penong	135		
Case contacts	301		
No. in postcode 5 690 (other than target suburbs above)	145	No denominator available	No separate estimate feasible
No. in postcode 5 680 (other than target suburbs above)	58		
**Total No. in wider target area (target suburbs and wider postcodes containing target suburbs)**	**3383**	**4000–4500**	**75–85%**
Total No. with suburb or postcode not stated or from another area	306	No denominator available	No separate estimate feasible

^a^ Population estimates used as the denominator for both suburb total and the total for suburb and wider postcodes containing target suburbs are those used in programme planning (4000–4500 persons). The lower bounds of the coverage estimates are based on the higher population estimate, and the higher bounds are based on the lower population estimate.

^b^ These are the household or household-like contacts vaccinated as part of immediate case follow-up.

No cases of IMD or meningococcal conjunctivitis caused by the quadrivalent vaccine serogroups were notified in either of the postcodes targeted by the programme during the duration of the vaccination campaign. Overall, there have been 11 cases of serogroup W meningococcal disease in SA since the end of the programme in June 2017 until the end of 2018, including two cases in the Ceduna area targeted by the vaccination programme: in July 2017, a case was notified in an adult male of non-Aboriginal background who had declined vaccination in Ceduna and whose three household contacts were also unvaccinated. In August 2018, another case was notified in an Aboriginal child who had not been born at the time of the vaccination programme and was a household contact of a previous Ceduna-area case. Fine typing for the first case in the post-vaccination period showed the strain to be of the same type as two of the pre-vaccination cases.

## Discussion and lessons learnT

The Ceduna community vaccination programme did not prevent the occurrence of further cases of IMD serogroup W in the area. Nevertheless, it demonstrated that community-wide vaccination is a useful public health response to a geographically limited outbreak of meningococcal disease. Despite the considerable logistical effort required, the programme reached up to 85% of the target population. Ongoing transmission was interrupted in the short-term, and given the high vaccination coverage, the large majority of residents can be assumed to have achieved immunity even if the programme may have failed to sufficiently reduce carriage rates and provide herd immunity in the medium term to long-term. Given the large knowledge gaps in the community, (*10*) the vaccination programme provided the additional benefit of educating the community about the signs and symptoms of IMD. As meningococcal ACWY vaccination has been funded under the National Immunization Program from July 2018 for infants and April 2019 for adolescents, there may not be a need for ad hoc community vaccination programmes in Ceduna and elsewhere in Australia unless an outbreak specifically affects cohorts who were not eligible for vaccination.

A post-response evaluation meeting identified three elements as critical to the successful implementation of the community vaccination programme. First, the response was locally driven, with a large number of health- and non-health-sector stakeholders involved in planning and implementing the programme. In particular, local community engagement ensured that clinics were appropriately staffed and vaccinations could be delivered in readily accessible community locations, such as the Ceduna Town Hall, which had the most regularly visited clinic. Second, the inclusion of a wide variety of stakeholders was supplemented with a clear response management structure, involving leads from all key agencies. The steering committee responded flexibly to external challenges, including the shortage of Nimenrix and initial confusion about the relation of the meningococcal W vaccination programme to a concomitant state-wide adolescent meningococcal B vaccination study. (*11*) Third, the community was generally receptive to the meningococcal W vaccination programme, which may have been helped by the involvement of local staff familiar with the programme and attuned to identifying local solutions. For instance, local Aboriginal health workers and Aboriginal health practitioners were able to assist Aboriginal participants in providing informed consent.

While more than 90% of vaccinations were administered to persons known to reside in the target postcodes, no proof of address was required. As a result, data completeness and quality for addresses was poor for a subset of records, and the majority of the remaining 10% for whom their postcode could not be determined are likely to also reside in the target area. Addresses given in surrounding areas, Greater Adelaide and other Australian jurisdictions suggest that a small number of persons vaccinated were not considered residents from an administrative point of view. As this may reflect travel patterns and community ties in a mobile, remote population, the vaccination of additional persons who may be de facto members of the target community is likely to have aided the response.

The programme encountered several logistical challenges. Estimating the quantity of vaccine required at different sites was challenging due to a lack of current population data at the town level and considerable fluctuation of population numbers in Aboriginal communities. Nevertheless, there was minimal wastage of vaccines: 79 vaccine doses needed to be discarded due to cold chain breaches at two separate sites, and there was no surplus vaccine because several other ACWY vaccination programmes were commenced simultaneously due to ongoing cases in other remote areas of SA. The vaccination programme at only one clinic had to be repeated due to an underestimation of population numbers at the site. Areas for improvement were identified with regard to several operational aspects of the response. These are related to the overarching recommendation to develop standard operating procedures for community-based interventions for outbreak response that can be adapted for state-wide use. They include:

standardizing provisions to allow staff to move between different regions of the CHSALHN and different departments of SA Health and avoiding the use of separate standing medication orders;
designating a single point of contact for clinical enquiries and decision support during the entire vaccination period;
streamlining media communications to reduce delays and lead-in time, including critical assessment of the value added by translations;
maximizing the use of community venues and offering extended and weekend opening times, resources permitting; and
improving data collection during the outbreak response, including recording Medicare numbers for the Australian Immunization Register and integrating clinical management software to enable follow-up of vaccinations.

## References

[1] Rosenstein NE, Perkins BA, Stephens DS, Popovic T, Hughes JM. Meningococcal disease. N Engl J Med. 2001 5 3;344(18):1378–88. 10.1056/NEJM200105033441807 11333996

[2] Heymann DL, editor. Control of communicable diseases manual. 19th ed. Washington (DC): American Public Health Association; 2015. 10.2105/CCDM.2745

[3] Wang B, Santoreneos R, Giles L, Haji Ali Afzali H, Marshall H. Case fatality rates of invasive meningococcal disease by serogroup and age: A systematic review and meta-analysis. Vaccine. 2019 5 9;37(21):2768–82. 10.1016/j.vaccine.2019.04.020 30987851

[4] Campbell H, Parikh SR, Borrow R, Kaczmarski E, Ramsay ME, Ladhani SN. Presentation with gastrointestinal symptoms and high case fatality associated with group W meningococcal disease (MenW) in teenagers, England, July 2015 to January 2016. Euro Surveill. 2016;21(12):30175. 10.2807/1560-7917.ES.2016.21.12.30175 27035055

[5] Christensen H, May M, Bowen L, Hickman M, Trotter CL. Meningococcal carriage by age: a systematic review and meta-analysis. Lancet Infect Dis. 2010 12;10(12):853–61. 10.1016/S1473-3099(10)70251-6 21075057

[6] Invasive meningococcal disease: CDNA national guidelines for public health. Canberra: Australian Government Department of Health; 2017. Available from: http://www.health.gov.au/internet/main/publishing.nsf/Content/cdna-song-IMD.htm, accessed 14 August 2018.

[7] Meningococcal disease (invasive): public dataset. Canberra: Australian Government Department of Health; 2018. Available from: http://www9.health.gov.au/cda/source/pub_menin.cfm, accessed 14 August 2018.

[8] Booy R. Recent and current epidemiology of invasive meningococcal disease – vaccines & vaccine policy. [slide presentation]. Sydney: National Centre for Immunisation Research and Surveillance; 2018. [cited 2018 August 14]. Available from: Available from https://immunisationcoalition.org.au/wp-content/uploads/2018/06/9.-Booy-meningococcal-talk.pdf

[9] Australian Technical Advisory Group on Immunisation. Australian Immunisation Handbook. Canberra: Australian Government Department of Health; 2018. Available from: https://immunisationhandbook.health.gov.au, accessed 14 August 2018.

[10] Wang B, Clarke M, Afzali HH, Marshall H. Community, parental and adolescent awareness and knowledge of meningococcal disease. Vaccine. 2014 4 11;32(18):2042–9. 10.1016/j.vaccine.2014.02.054 24593997

[11] Marshall HS, McMillan M, Koehler A, Lawrence A, MacLennan JM, Maiden MCJ, et al. B Part of It protocol: a cluster randomised controlled trial to assess the impact of 4CMenB vaccine on pharyngeal carriage of *Neisseria meningitidis* in adolescents. BMJ Open. 2018 7 10;8(7):e020988. 10.1136/bmjopen-2017-020988 29991629 PMC6082482

